# P-90. Microbiology of Spinal Fusion Infections: An Analysis of the National Healthcare Safety Network Database

**DOI:** 10.1093/ofid/ofaf695.319

**Published:** 2026-01-11

**Authors:** Molly Lindstrom, Benjamin Langworthy, Jonathan Sembrano, James Glover, Don Bambino Geno Tai

**Affiliations:** University of Minnesota, Minneapolis, MN; University of Minnesota, Minneapolis, MN; University of Minnesota, Minneapolis, MN; University of Minnesota, Minneapolis, MN; University of Minnesota, Minneapolis, MN

## Abstract

**Background:**

Understanding the current microbial causes of surgical site infections (SSIs) following spinal fusion surgeries is essential for guiding prevention strategies and empiric antibiotic choices. This study aimed to characterize the microbiology of these infections and identify risk factors associated with Gram-negative (GN) infections.

**Table 1:**
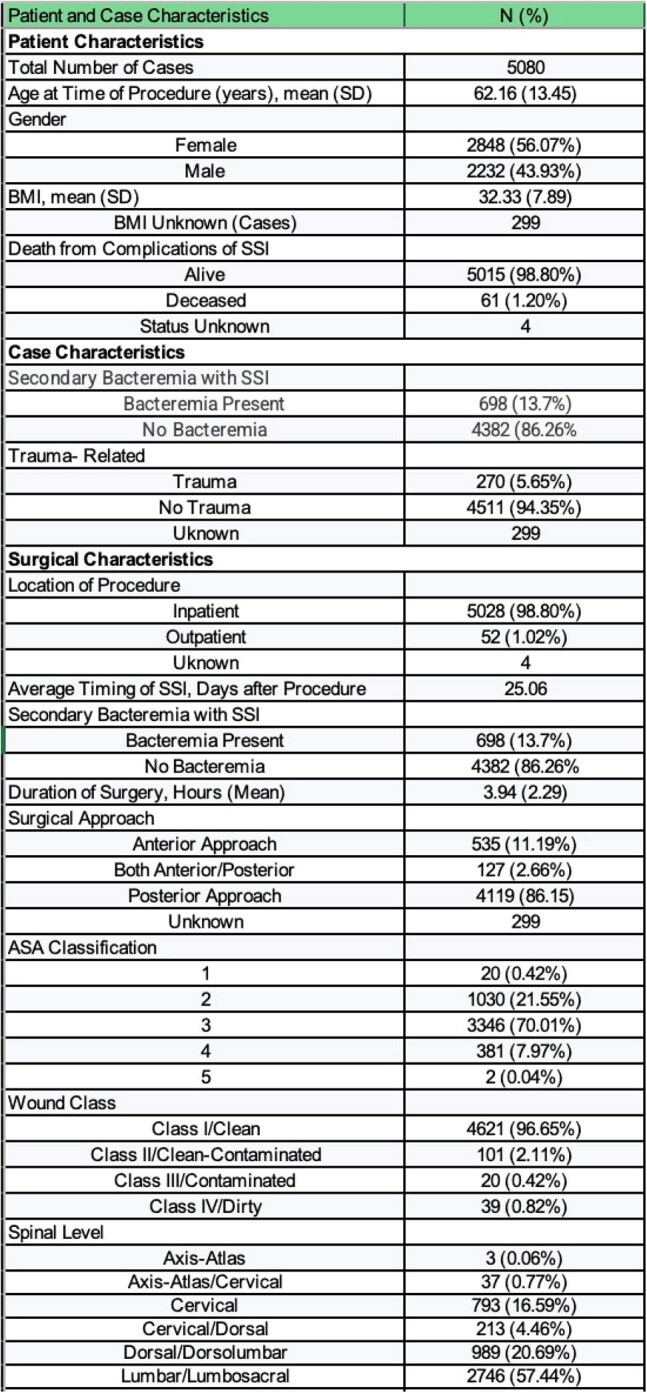
Summary of Patient and Case Demographics

All cases of spinal fusion SSIs that were reported to the NHSN database between 2021 and 2022 were analyzed. Any cases involving SSI that had a known infection at the time of diagnosis of SSI were excluded.

**Table 2:**
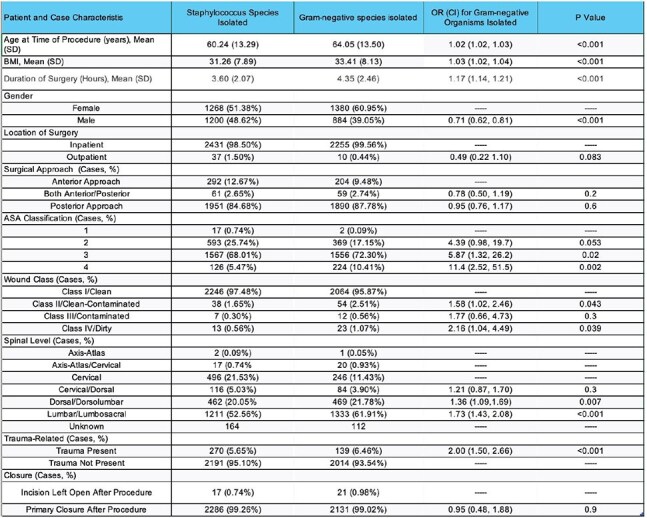
Risk Factors for Gram-Negative Organism Infection in Spinal Fusion SSI

We analyzed both patient and surgical factors compared to the number of gram-negative organisms identified in cultures for SSIs. Odds ratios were calculated using Staphylococcus species as a comparator group to identify what characteristics provided a higher risk of GN involvement in SSI. Table 2 shows the number of Staphylococcus species and GN species identified in culture per patient characteristic and surgical characteristic, along with the odds ratio and confidence intervals for GN species involvement for each characteristic.

**Methods:**

We analyzed spinal fusion SSIs reported to the National Healthcare Safety Network (NHSN) between 2021 and 2022. Cases with infections present at the time of surgery were excluded. We used staphylococcal infections as a comparator group to GN infections and performed a multinomial regression analysis to assess associations between microbial etiology and patient or surgical factors.

**Results:**

The study included 5,080 cases. The median patient age was 62 years, and 44% were male. Monomicrobial infections accounted for 61% of cases; 18% were polymicrobial. The most common organism isolated was *Staphylococcus aureus* (31%). GN organisms were isolated in 32% of infections.

GN infections were more likely to occur in older patients, females, and those with higher BMI. Patients with ASA class 3 or 4 also had an increased risk. Compared to cervical spine procedures, the likelihood of GN infections increased with more caudal surgical sites: 61.9% of GN bacterial infections were from lumbar SSIs, and only 11.4% were from cervical cases. Trauma-related surgeries were twice as likely to result in GN infections relative to *Staphylococcus*-only infections.

**Conclusion:**

While *Staphylococcus* species are the most common organisms involved in spinal fusion SSI, GN organisms are also frequently involved. GN organisms are more likely to be involved in infections with caudal surgical sites, older patients, females, elevated BMI, trauma-related cases, and ASA class 3 or 4. Current SSI prophylactic regimens have limited spectrums of GN coverage, so further investigation to evaluate expanded prophylactic antibiotic coverage in cases that are high-risk for GN involvement is needed. Similarly, empiric antibiotic coverage for septic patients should be expanded to include GN coverage when these risk factors are present.

**Disclosures:**

Jonathan Sembrano, MD, Medtronic: Grant/Research Support|Medtronic: Fellowship support|NuVasive: Grant/Research Support|SI-Bone: Fellowship support

